# Geometrically Controlled Asymmetric Division of CD4+ T Cells Studied by Immunological Synapse Arrays

**DOI:** 10.1371/journal.pone.0091926

**Published:** 2014-03-14

**Authors:** Hong-Ryul Jung, Kwang Hoon Song, John T. Chang, Junsang Doh

**Affiliations:** 1 School of Interdisciplinary Bioscience and Bioengineering (I-Bio), Pohang University of Science and Technology (POSTECH), Pohang, Korea; 2 Department of Mechanical Engineering, Pohang University of Science and Technology (POSTECH), Pohang, Korea; 3 Department of Medicine, University of California San Diego, La Jolla, California, United States of America; University of North Carolina at Chapel Hill, United States of America

## Abstract

Similar to stem cells, naïve T cells undergo asymmetric division following activation. While asymmetric division of T cells has been shown to be an important mechanism for the generation of lymphocyte fate diversity during immune responses, key factors that influence whether T cells will undergo symmetric or asymmetric divisions are not completely understood. Here, we utilized immunological synapse arrays (ISAs) to begin to dissect mechanisms of asymmetric T lymphocyte division. ISAs are protein micropatterned surfaces composed of two segregated regions, activation sites and adhesion fields. Activation sites are small spots presenting activation signals such as anti-CD3 and anti-CD28, and adhesion fields are the remaining regions surrounding activation sites immobilized with interintercel adhesion molecule 1 (ICAM-1). By varying the size and the distance between the activation sites and measuring the incidence of asymmetric cell divisions, we found that the distance between activation sites is an important regulator of asymmetric division. Further analysis revealed that more symmetric divisions occurred when two nascent daughter cells stably interacted with two distinct activation sites throughout and following cytokinesis. In contrast, more asymmetric divisions occurred when only one daughter cell remained anchored on an activation site while the other daughter became motile and moved away following cytokinesis. Together, these results indicate that TCR signaling events during cytokinesis may repolarize key molecules for asymmetric partitioning, suggesting the possibility that the density of antigen presenting cells that interact with T cells as they undergo cytokinesis may be a critical factor regulating asymmetric division in T cells.

## Introduction

During immune responses, T cells activated by recognizing their target antigens presented by antigen presenting cells (APCs) undergo clonal expansion to increase number of T cells reacting to invading microbial pathogens. At the same time, proliferating T cells differentiate into various subsets of effector and/or memory T cells to efficiently mount both acute and recurrent immune responses to infection [1]–[3]. Although the mechanisms that allow a single T cell to generate phenotypically distinct subsets of T cells remain incompletely understood [4]–[7], asymmetric division has been shown to be one of the mechanisms that generate this diversity by regulating effector/memory formation of CD8+ T cell and differentiation of CD4+ T cells [8]–[10].

In lymph nodes, rapidly migrating T cells slow down their motility when they encounter dendritic cells (DCs) presenting their target antigens, cease to stably interact with DCs for several hours, regain motility, and undergo cell division [11]–[13]. Stable interactions between T cells and DCs are mediated by the molecular interaction of lymphocyte function-associated antigen 1 (LFA-1) on T cells and intercellular adhesion molecule 1 (ICAM-1) on DCs [14]. T cell receptor (TCR) signaling triggered by antigenic peptide loaded on major histocompatibility complex (MHC) of DCs activates LFA-1 to induce strong adhesion of T cells on DCs [15]. At the interfaces between stably interacting T cells and APCs, receptors, signaling molecules, and adapter proteins are polarized and assembled into distinct supramolecular struetures, the so-called immunological synapses (ISs) [16], [17]. Key signaling molecules such as TCR and CD28 accumulate at the central area of the IS while the adhesion molecule LFA-1 is enriched at the periphery of the IS [18]. Formation of the IS has been suggested to be important for setting up asymmetric T cell division, but key factors dictating whether T cells will undergo symmetric or asymmetric division have not been investigated [8], [19].

**Figure 1:**
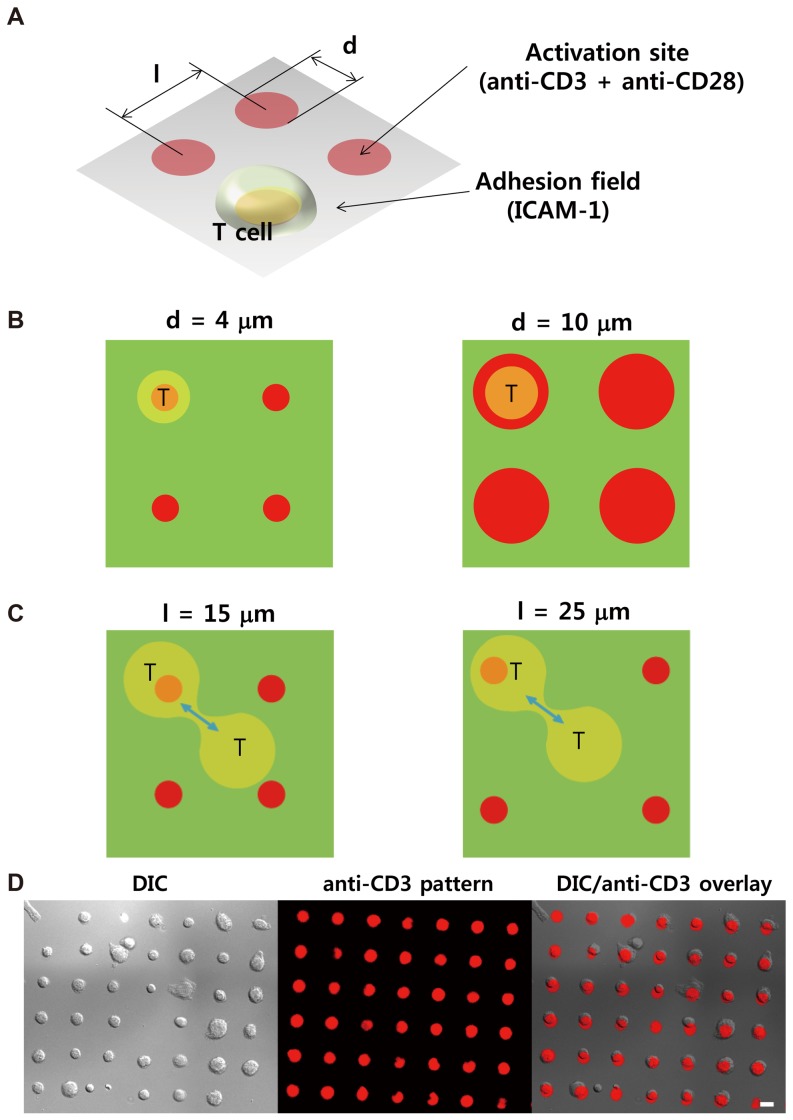
Design and fabrication of the immunological synapse arrays (ISAs). A. Schematic illustration of an ISA. **B.** Schematic illustration of T cells on ISAs with various diameters of the activation sites. **C.** Schematic illustration of dividing T cells on ISAs with various distances between the activation sites. **D.** Representative images of T cells on ISAs. An ISA with d = 10 μm and l = 25 μm was used, and images were acquired 4 h after seeding. Alexa-647-labeled anti-CD3 was used to visualize the activation sites. Differential interference contrast (DIC) image (left), fluorescence image of anti-CD3-Alexa-647 (middle), and overlay (right). Scale bar: 10 μm.

Synthetic surfaces have been useful in addressing fundamental questions in T cell activation and immune synapse formation [20], [21]. In particular, immunological synapse arrays (ISAs) [22], protein micropatterned surfaces presenting key molecules for T cell activation developed to study the effect of the synapse structure on T cell activation, can be useful to systematically study asymmetric T cell division. As schematically shown in Fig. 1A, the ISAs are composed of two distinct regions: activation sites and adhesion fields. Activating signals presenting central region of the IS were immobilized in the activation sites, while the adhesion molecule ICAM-1 was attached in the adhesion field. Presentation of ICAM-1 in the adhesion field has dual roles: it may support a ‘stop’ signal triggered by TCR signaling at the activation sites, but it may also provide a ‘go’ signal to trigger motility of T cells [23]. We and others have previously shown that T cells initially land on the adhesion field polarize and migrate until they encounter the activation sites [22], [24]. When they contact the activation sites, they halt and stably interact with the activation sites for several hours. They subsequently regain motility and leave the activation sites, migrate to other activation sites and remain there for approximately 1–2 h, and eventually undergo cell division [22], [25].

In this study, motivated by the observation that dynamics of T cells interacting with ISAs is similar to dynamics of T cells within lymph nodes, we utilized the ISAs to dissect key factors regulating asymmetric T cell division. By systematically varying the size of and distance between activation sites, we found that the distance between activation sites is an important factor regulating for asymmetric T cell division. Moreover, additional analysis revealed that interactions between the activation sites and nascent daughter cells during cytokinesis were important for asymmetric division of T cells.

## Materials and Methods

### Cells and reagents

Using a CD4+ T cell negative selection kit (StemCell Technologies Inc.), CD4+ T cells were purified from lymph nodes and spleens of 6–8 week old C57BL/6 mice, which were bred in the animal care facility in POSTECH Biotech Center (PBC). All experiments involving mice were approved by the Institutional Animal Care and Use Committee in PBC. Anti-CD3 (clone: 2C11) was purchased in a large scale from BioXCell and custom labeled with N-hydroxysuccinimide (NHS)-activated forms of fluorescein (Pierce), Alexa Fluor 555 (Invitrogen), and Alexa Fluor 647 (Invitrogen) according to the instructions from the vendors. Anti-CD28 (clone: 37.51) was purchased from BioXCell, anti-TCRβ-FITC (clone: H57-597), anti-T-bet (clone: eBio4B10), and isotype control IgG were purchased from eBioscience, anti-tubulin-Cy3 (clone: TUB 2.1) was purchased from Sigma-Aldrich, anti-PKCζ was purchased from Abcam, and ICAM-1/Fc was purchased from R&D systems.

### Fabrication of immunological synapse arrays (ISAs)

ISAs were fabricated by microcontact printing as previously described [24]. PDMS stamps for microcontact printing were fabricated by pouring Sylgards 184 (Dow Corning, Korea) precursor mixtures (base:curing agent  = 10∶1 ratio) on silicon masters, which were fabricated by standard photolithography and deep reactive ion etching, and curing for 2 h at 80°C. No. 1 Glass coverslips of 18 mm diameter (Marienfeld) were cleaned by treating with air plasma (200 ∼ 500 W, Femto Science, Korea) for 1 min. A drop of anti-CD3/CD28 solution in phosphate buffered saline (PBS) was applied on a PDMS stamp, incubated for 30 min, briefly rinsed with deionized (DI) water, and brought into a conformal contact with the cleaned coverslips for 1 min. Finally, the anti-CD3/CD28-printed surfaces were coated with 5 μg/ml of ICAM-1/Fc for 2 h.

### Immunofluorescence microscopy of T cells on the ISAs

CD4+ T cells suspended in RPMI 1640 medium (Life Technologies) supplemented with 10% of FBS (Life Technologies), 100 U/ml of penicillin, 100 mg/ml of streptomycin (Life Technologies), and 0.1% of beta-mercaptoethanol (Sigma) were seeded on the ISAs with ∼ 10^6^ cells/ml of density. ISAs containing T cells were then incubated for 32 h in a tissue culture incubator maintaining 5% CO_2_ and 37°C, fixed with 4% paraformaldehyde for 15 min at room temperature, washed with PBS, and stained with fluorescent-labeled antibodies. A modified Zeiss Axio Observer.Z1 epifluorescence microscope with a 40X objective lens (Plan-Neofluar, NA = 1.30) and a Roper Scientific CoolSnap HQ CCD camera was used for imaging. XBO 75 W/2 Xenon lamp (75 W, Osram) and DAPI (EX. 365, BS 395, EM BP 445/50), eGFP (EX BP 470/40, BS 495, EM BP 525/50), Cy3 (EX BP 550/25, BS 570, EM BP 605/70), Cy5 (EX BP 620/60, BS 660, EM BP 770/75) excitation/emission filter sets were used for illumination and fluorescence imaging. The microscope was automatically controlled by Axiovision 4.6, (Carl Zeiss) and acquired images were analyzed and processed with Metamorph (Universal Imaging, Molecular Devices) or ImageJ (NIH). Fluorescence images of fixed and stained T cells on the ISAs were acquired by optical sectioning (8 individual planes, 1 μm apart) and analyzed by integration through z-sections.

### Live cell imaging of T cells on the ISAs

The ISAs containing T cells loaded in Chamlide chambers (Live Cell Instrument, Korea) were mounted on the microscope stage equipped with a Chamlide TC incubator system maintaining 37°C and 5% CO_2_ (Live Cell Instrument, Korea). Time-lapse microscopy was initiated with images recorded at intervals of 30 s for 1.5 h. Acquired time-lapse images were analyzed with Metamorph or Image J. To correlate patterns of cytokinesis and asymmetric division, T cells on ISA were fixed immediately after live cell imaging while the ISAs were mounted on the microscope stage, stained for TCR, and immunofluorescence microscopy was performed as described above.

## Results and Discussion

### Design and fabrication of the immunological synapse arrays (ISAs)

ISAs with various diameters of the activation sites (d) and inter-activation site distances (l) were fabricated as schematically shown in Fig. 1A. Activation signals such as anti-CD3 and anti-CD28 were presented in the activation sites while adhesion molecule ICAM-1 was immobilized in the adhesion field [22], [24]. To test the effect of modulating the size of the activation sides, we used two different diameters of activation sites, 4 μm and 10 μm. Since the average diameter of primary CD4+ T cells isolated from spleens and lymph nodes of mice is about 7 μm [26], T cells interacting with activation sites with a 4 μm diameter will receive both anti-CD3/CD28 signals and ICAM-1 signals in a spatially segregated manner (Fig. 1B, left panel). In contrast, T cells interacting with activation sites with a 10 μm diameter will mostly receive anti-CD3/CD28 signals, at least when activation is initiated (Fig. 1B, right panel). However, following activation, the diameter of T cells may become greater than 10 μm [26], so CD4+ T cells are likely to receive ICAM-1 signals at the periphery of the activation sites. Two different inter-activation site distances of 15 μm and 25 μm were selected to control interactions between the activation sites and nascent daughter cells undergoing cytokinesis (Fig. 1C). With the smaller inter-activation site distance, two nascent daughter cells undergoing cytokinesis will have a greater chance to interact with two distinct activation sites simultaneously compared with the greater inter-activation site distance. Primary murine CD4+ T cells purified from spleens and lymph nodes of C57BL/6 mice were seeded on the ISAs. As previously described [22], [24], when T cells encounter the activation sites, they halted and stably interacted with the activation sites for several hours. As a result, within 1 h of seeding, near-perfect single cell arrays of T cells were formed (Fig. 1D).

### Assessment of asymmetric distribution of molecules in nascent daughter cells on ISAs

**Figure 2:**
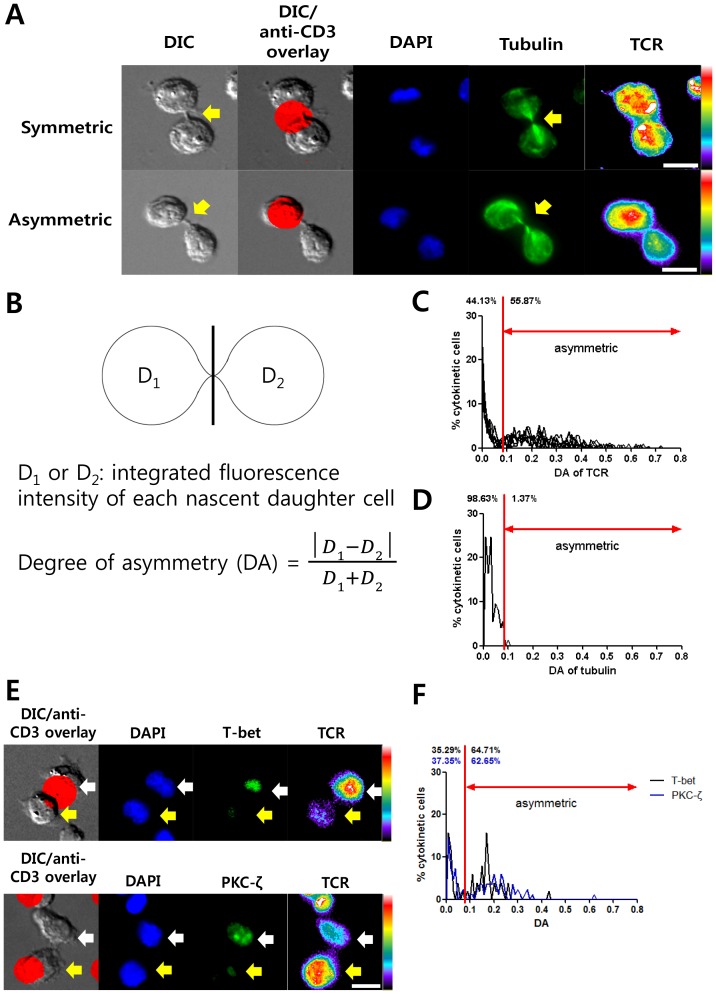
Assessment of asymmetric distribution of molecules in nascent daughter cells on ISAs. A. Representative images of T cells undergoing cytokinesis. ISAs with d = 10 μm and l = 25 μm was used, and images were acquired 32 h after seeding. For fluorescence images, z-stack images were integrated into a single plane and displayed. For TCR images, fluorescence intensities were visualized by rainbow pseudo color to clearly show intensity differences between nascent daughter cells. Scale bar: 10 μm. **B.** Scheme of quantitative evaluation of degree of asymmetry (DA). **C.** DA value distribution of TCR in two nascent daughter cells (n = 849). **D.** DA value distribution of tubulin in two nascent daughter cells (n = 75). **E.** Representative images of PKC-ζ and T-bet in nascent daughter cells (left) and DA value distributions of PKC-ζ (n = 50) and T-bet (n = 80). Scale bar: 10 μm.

To assess the incidence of asymmetric division, CD4+ T cells were seeded on different types of ISAs, fixed after 32 h, stained with DAPI and fluorophore-conjugated anti-TCR and anti-tubulin antibodies, and imaged by fluorescence microscopy. By using a motorized stage, entire ISAs were examined and T cells undergoing cytokinesis were first identified based on their morphology in differential interference contrast (DIC) and further confirmed by tubulin fluorescence images. First, two neighboring cells connected by thin fibrous structures were selected by examining DIC images (yellow arrows of DIC images in Fig. 2A). This morphology is one of the characteristics of cells undergoing cytokinesis, but two non-dividing but adjoining T cells may also form such structures via membrane nanotubules for intercellular communication [27]. While membrane nanotubules connecting two adjoining T cells contain little amount of tubulin [27], thin fibrous structures formed between two nascent daughter cells are enriched with tubulins due to compaction of central spindles [10], [28]. Therefore, tubulin staining was examined to see whether tubulins were accumulated in the fibrous structures or not. Importantly, more than 95% of two neighboring T cells connected with the thin fibrous structures exhibited strong accumulation of tubulins in the fibrous structures (n = 68, yellow arrows of tubulin images in Fig. 2A), suggesting that morphology based on DIC images along was sufficient to identify cytokinesis in our experimental settings. Large area automated imaging was necessary for the unbiased sampling because only small fractions of T cells (1∼2%) were undergoing cytokinesis. Typically, about 30 T cells undergoing cytokinesis phase could be detected per ISA.

Among cytokinetic cells, T cells undergoing asymmetric division were identified by quantitatively examining the distribution of the T cell receptors (TCRs) as schematically shown in Fig. 2B. Integrated fluorescence intensity of each nascent daughter cell (D_1_ or D_2_) was measured, and the degree of asymmetry (DA) defined by the equation shown in Fig. 2B was calculated. A similar methodology was previously used to quantitatively evaluate asymmetric distribution of molecules in T cells during cell division [9]. If two nascent daughter cells contained similar amounts of TCRs, or if T cells were undergoing a symmetric cell division, the DA value would be close to 0. To determine a threshold DA value with which to score asymmetric cell divisions, DA values of TCRs were calculated from a large number of cytokinetic T cells (n = 849) and the distribution of DA values were plotted in Fig. 2C. Two separate populations were clearly distinguishable with a DA value of 0.08 as the cut-off, indicating that DA = 0.08 can be used for a threshold value to identify asymmetric divisions. In sharp contrast, when we examined DA values of tubulin, which is known to distribute symmetrically [8]–[10], only one population with DA values less than 0.1 was detected (Fig. 2D). Other markers that have been previously reported to be associated with asymmetric division of T cells such as T-bet [10] and the ζ form of protein kinase C (PKC-ζ) [8]–[10] were examined along with TCR (Fig. 2E). Similar to TCR, two clear populations could be distinguished with a DA value of 0.08 (Fig. 2F). Taken together, quantitative assessment of the asymmetric distribution of molecules using our methodology agreed well with the incidence of asymmetry in previous reports [8]–[10].

### Effect of the ISA geometry on asymmetric T cell division

To assess the effects of ISA geometry on asymmetric T cell division, we generated ISAs with four different geometries, as schematically shown in Fig. 1A. For the bulk of experiments, 10 μg/mL of anti-CD3 and 10 μg/mL of anti-CD28 were used for microcontact printing. Relatively high concentrations of antibodies were selected to maximize polarization of T cells and frequencies of cytokinesis [17], [29], [30]. T cells on activation sites with a diameter of 10 μm should receive 6.25 times more activation signals than T cells on activation sites with a diameter of 4 μm if identical amounts of antibodies are used. Thus, to provide identical amount of activation signals to 4 μm activation sites, the concentrations of anti-CD3 and anti-CD28 antibodies on 10 μm activation sites would need to be diluted 6.25-fold. Anti-CD3 and anti-CD28 antibodies were therefore diluted 6.25 times to 1.6 μg/mL, and 16.8 μg/mL of isotype control antibody was added to the solution to keep total concentration of antibody 20 μg/mL. In this way, densities of anti-CD3 and anti-CD28 printed on the surfaces would be proportional to the concentrations of anti-CD3 and anti-CD28, as previously demonstrated [24].

**Figure 3:**
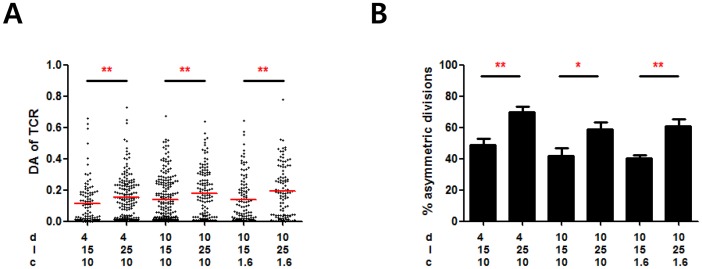
Effect of the ISA geometry on asymmetric division of T cells. A. DA values of nascent daughter cells on different types of the ISAs. d: activation site diameter, l: inter-activation site distance, and c: concentration of antibodies. (Mann-Whitney U-test, **p<0.01). **B.** Percentage of asymmetric cell division on different types of the ISAs. Data are means of 3 independent experiments and error bars are standard error of means. In each experiment, about 30 nascent daughter cells were assessed. (t-test, *p<0.05, **p<0.01).

T cells seeded on different types of ISAs described above were fixed 32 h after seeding, stained with anti-TCR-FITC, and examined by immunofluorescence microscopy. DA for the TCR of individual T cells on each type of ISA was measured and plotted in Fig. 3A. The incidence of asymmetric divisions was quantitated by counting T cells with a DA of TCR greater 0.08 and shown in Fig. 3B. Both the DA values and the incidence of asymmetric T cell divisions were significantly influenced by the distance between the activation sites, but did not depend on the amount of activating signals or the size of the activation sites. Thus, a higher incidence of asymmetric cell division was observed with larger inter-activation site distances.

### Correlation between asymmetric division and relative positions of daughter cells

**Figure 4:**
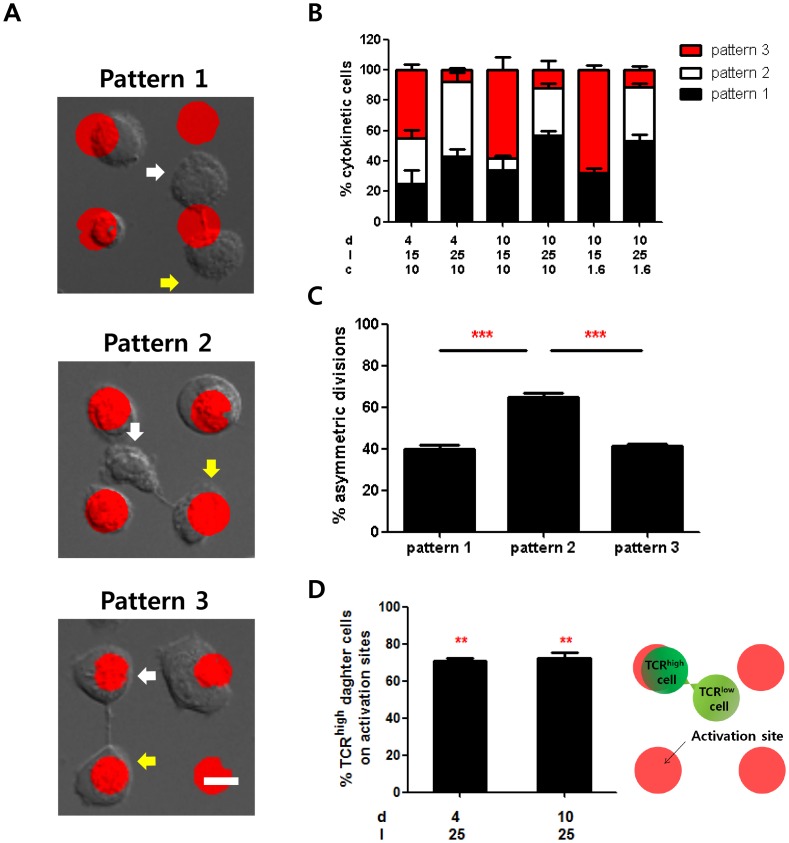
Correlation between relative positions of daughter cells and asymmetric division. A. Representative images of different patterns of T cells undergoing cytokinesis. Pattern 1: two nascent daughter cells contacting the same activation site. Pattern 2: Only one nascent daughter cell contacting an activation site. Pattern 3: Two nascent daughter cells contacting two distinct activation sites. Scale bar: 10 μm. **B.** Percentage of each pattern of T cells on different types of ISAs. **C.** Percentage of T cells in each cytokinetic pattern undergoing asymmetric division. **D.** Frequency of TCR^high^ daughter cells locating on activation sites. Among asymmetrically dividing T cells in pattern 2, positions of daughter cells expressing higher levels TCRs were counted and quantified. For statistical analysis, mean values were compared with 50. Data are means of 3 independent experiments and error bars are standard error of means. In each experiment, about 30 nascent daughter cells were assessed. (t-test, *p<0.05, **p<0.01, ***p<0.001).

We further analyzed the correlation between asymmetric division of T cells and positions of nascent daughter cells with respect to the activation sites. Dividing T cells were observed to fall into one of three possible patterns, as shown in Fig. 4A: (1) two nascent daughter cells contacting the same activation site (pattern 1, top panel of Fig. 4A), (2) only one nascent daughter cell contacting an activation site with the other nascent daughter cell staying in the adhesion field (pattern 2, middle panel of Fig. 4A), or (3) both nascent daughter cells contacting two different activation sites (pattern 3, bottom panel of Fig. 4A). The percentage of each pattern influenced by different types of ISAs was quantitated and shown in Fig. 4B. Overall, higher percentages of patterns 1 and 2 were observed for T cells on the ISAs with inter-activation site distances of 25 μm; conversely, higher percentages of pattern 3 were observed for T cells on ISA with inter-activation site distances of 15 μm, regardless of the activation site diameter and stimulating antibody concentration. In addition, when we measured the incidence of asymmetric division occurring with each pattern of cytokinesis, we observed a 1.5 fold higher incidence of asymmetry for pattern 2 compared to patterns 1 or 3. (Fig. 4C). These results indicate that asymmetric division more frequently occurred with higher inter-activation distances, presumably because dividing T cells were less likely to encounter neighboring activation sites during cytokinesis. In addition, among asymmetrically dividing T cells exhibiting pattern 2, the majority of TCR^high^ cells were contacting the activation sites (Fig. 4D) regardless of the sizes of the activation sites. These results indicate that during cytokinesis, TCRs in each daughter cell can be polarized by the signals at the activation sites. Therefore, if two daughter cells contact activation sites simultaneously (pattern 1 and 3), they will more likely to undergo symmetric cell division. Conversely, if only one daughter cell remains in contact with an activation site (case 2), TCRs will remain polarized to the daughter cell contacting the activation site, resulting in asymmetric cell division.

### Dynamics of T cells undergoing cytokinesis on ISAs

**Figure 5:**
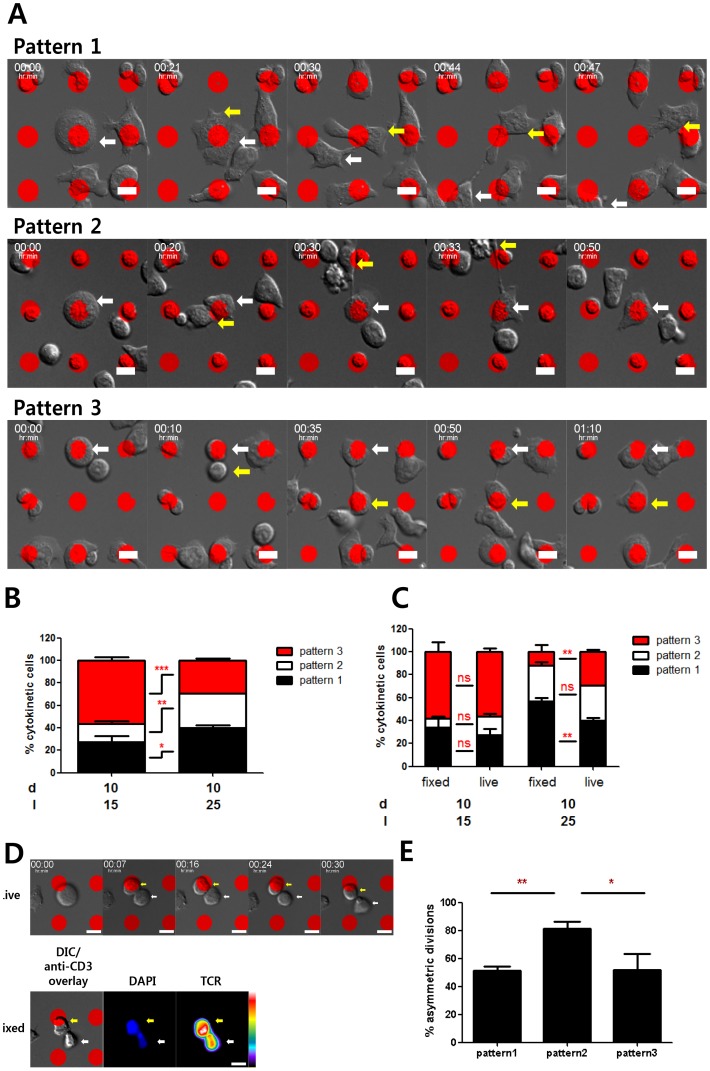
Dynamics of dividing T cells on ISAs. A. Representative time-lapse images of different patterns of cytokinesis exhibited by dividing T cells. Pattern 1: Cytokinesis occurred with the cell contacting an activation site, with both nascent daughter cells moving away from the activation site following cytokinesis. Pattern 2: One daughter cell remained anchored on an activation site and stably interacted with the activation site throughout cytokinesis while the other daughter cell was more motile and moved away following cytokinesis. Pattern 3: Two nascent daughter cells stably interacted with two distinct activation sites during and following cytokinesis. Scale bar: 10 μm. **B.** Percentage of each cytokinesis pattern resulting from different types of ISAs. (Two-way ANOVA-test with Bonferroni post-tests, *p<0.05, **p<0.01, ***p<0.001). **C.** Comparision between cytokinesis patterns classified by fixed cell imaging (Fig. 4B) and live cell imaging (Fig. 5B). (Two-way ANOVA-test with Bonferroni post-tests, ns: not significant, **p<0.01). **D.** Representative time-lapse images of T cells undergoing cytokinesis (upper) and their TCR/DAPI staining at the end of time-lapse imaging (lower). T cells on ISAs were fixed immediately after time-lapse imaging, stained with anti-TCR and DAPI, and examined by immunofluorescence microscopy. **E.** Percentage of T cells in each cytokinetic pattern undergoing asymmetric division was determined. Cytokinesis patterns were classified by live cell imaging and asymmetric division was identified by immunofluorescence microscopy of cells fixed immediately after time-lapse imaging. Data are means of 5 independent experiments and error bars are standard error of means. In each experiment, about 15 nascent daughter cells were assessed. (t-test, *p<0.05, **p<0.01).

Thus far we have only shown T cells fixed on the ISAs. Cytokinesis of T cells is a highly dynamic process occurring over several hours [11], [22], so assessments based on the fixed snapshots may be misleading. To overcome this issue, we performed live cell imaging of T cells on the ISAs. The ISAs were seeded with T cells, incubated for 32 h in a tissue culture incubator, and mounted on a microscope stage equipped with a Chemlide TC incubator system maintaining 37°C temperature and 5% CO_2_. As mentioned above, only a small fraction of T cells were undergoing cell divisions at this time point. To increase the probability of observing T cell division on the ISAs, we first scanned the entire ISAs using a motorized stage, and then selected stage positions containing T cells in mitosis. Every 30 sec, DIC images and fluorescence images of the activation sites were acquired from the selected stage positions. The number of stage positions imaged in a time-lapse imaging was limited by time-interval, so about 30 stage positions were imaged and about 15 cytokinetic T cells were observed per each time-lapse imaging experiment. Throughput of live cell imaging was extremely low compared with fixed cell analysis: only one ISA can be examined with each experiment, and the frequency of observing cytokinetic T cells in an ISA was also low. Therefore, only selected sets of ISAs among six different types of ISAs used for fixed T cell analysis were used for live cell imaging. Since asymmetric division of T cells was mostly affected by the distances between activation sites, two different types of ISAs with the identical activation site diameter of 10 μm with different inter-activation site distances (15 μm and 25 μm) were fabricated and used. Time-lapse images of dividing T cells were carefully examined and classified into three patterns depending on the interactions between daughter cells and the activation sites during and after cytokinesis: (1) Cytokinesis occurring with both daughter cells contacting the same activation site, then moving away from the activation site after cytokinesis (pattern 1, top panel of the Fig. 5A and Movie S1). (2) One daughter cell anchored on an activation site and stably interacting with the activation site throughout cytokinesis with the other daughter cell moving away following cytokinesis (pattern 2, middle panel of the Fig. 5A and Movie S2)). (3) Two daughter cells stably interacting with two distinct activation sites during and following cytokinesis (pattern 3, bottom panel of the Fig. 5A and Movie S3). Patterns 1, 2, and 3 described in Fig. 5 appeared to match with patterns 1, 2, and 3 described in Fig. 4, respectively. Time-lapse images of T cells on each type of ISA were manually examined, categorized into each case, and plotted in Fig. 5B. The frequencies of patterns 1 and 2 increased and frequency of pattern 3 decreased as inter-activation site distance increased, similar to the trends observed using the fixed approach (Fig. 4B). Although the incidence of pattern 1 and pattern 3 divisions observed differed with the live and fixed imaging approaches, we demonstrate that pattern 2 divisions, which exhibited the highest incidence of asymmetric division in fixed cell analysis (Fig. 4C), could be accurately identified by either approach (Fig. 5C). To test whether divisions identified as pattern 2 by live cell imaging were indeed mostly asymmetric, as assessed by a fixed cell imaging approach, cells were fixed immediately after live cell imaging and stained for TCR while the ISA used for the live cell imaging was still mounted on the microscope stage. In this way, the distribution of TCR at the end of the live cell imaging could be visualized (Fig. 5D), thereby allowing us to correlate each pattern of cytokinesis in live cell imaging with symmetric vs. asymmetric divisions. As shown in Fig. 5E, a significantly higher incidence of asymmetric division was observed in pattern 2 compared to other patterns, which agreed well with the results based on fixed cell analysis (Fig. 4C). Live cell imaging of cytokinec T cells expressing TCR-GFP fusion protein would serve to confirm these results, but due to technical difficulties such as low transfection efficiency of naïve T cells and low frequencies of cytokinesis, we were unable to perform such experiments. Taken together, these data suggest that strong TCR stimulation encountered by one nascent daughter cell during cytokinesis might trigger polarization of TCR to that side, resulting in asymmetric division of T cells.

Previous research has suggested that LFA-1-mediated stable interactions between T cells and DCs triggered by strong TCR signaling are critical for stabilizing polarization of key molecules in asymmetric division of T cells [8], [10], [31]. However, dynamics of T cells in cytokinesis has not been previously considered. In this study, by varying distances between activation sites and carefully examining the interaction of dividing daughter cells with activation sites and determining the incidence of asymmetric cell divisions, we have provided evidence that polarization induced by initial stable synapse formation can be altered during cytokinesis. The distance between activation sites in ISAs may be translated into the density of APCs presenting antigens specific for a dividing T cell during immune response *in vivo*. It is possible that high density of APCs triggering TCR signals of a given T cell may trigger the onset of autoimmunity because self-antigens are the most abundant antigens. Since asymmetric division of T cells has been shown to regulate the generation of memory T cells [8], a higher density of APCs presenting a specific antigen might suppress asymmetric division, potentially preventing the generation of memory T cells specific for self-antigens and in so doing, avert autoimmunity.

In summary, we studied asymmetric cell division of CD4+ T cells using ISAs, and observed that the distance between the activation sites was an important regulator of asymmetric T cell division. We also found evidence that TCR signaling during cytokinesis repolarized key molecules for asymmetric division, potentially adding another checkpoint for asymmetric cell division. Together, these results provide new insights into the mechanisms underlying asymmetric T lymphocyte division.

## Supporting Information

Movie S1 **Representative movie of a T cell undergoing cytokinesis with pattern 1 in** **Fig. 4A** **.** Both nascent daughter cells are moving away from the activation site following cytokinesis. Time stamp  =  hr:min, Scale bar  =  10 μm.
(MOV)

Movie S2 **Representative movie of a T cell undergoing cytokinesis with pattern 2 in** **Fig. 4A** **.** One daughter cell remains anchored on an activation site and stably interacts with the activation site throughout cytokinesis while the other daughter cell is more motile and move away following cytokinesis. Time stamp  =  hr:min, Scale bar  =  10 μm.
(MOV)

Movie S3 **Representative movie of a T cell undergoing cytokinesis with pattern 3 in** **Fig. 4A** **.** Two nascent daughter cells stably interact with two distinct activation sites during and following cytokinesis. Time stamp  =  hr:min, Scale bar  =  10 μm.
(MOV)

## References

[1] Williams MA, Bevan MJ (2007) Effector and memory CTL differentiation. Annual Review of Immunology 25: 171–192. 10.1146/annurev.immunol.25.022106.141548 17129182

[2] Zhu J, Yamane H, Paul WE (2010) Differentiation of effector CD4+ T cell populations. Annual Review of Immunology 28: 445–489. 10.1146/annurev-immunol-030409-101212 PMC3502616 20192806

[3] Lanzavecchia A, Sallusto F (2005) Understanding the generation and function of memory T cell subsets. Current Opinion in Immunology 17: 326–332. 15886125 10.1016/j.coi.2005.04.010

[4] Stemberger C, Huster KM, Koffler M, Anderl F, Schiemann M, et al (2007) A Single Naive CD8+ T Cell Precursor Can Develop into Diverse Effector and Memory Subsets. Immunity 27: 985–997. 18082432 10.1016/j.immuni.2007.10.012

[5] Gerlach C, Van Heijst JWJ, Swart E, Sie D, Armstrong N, et al (2010) One naive T cell, multiple fates in CD8+ T cell differentiation. Journal of Experimental Medicine 207: 1235–1246. 20479114 10.1084/jem.20091175 PMC2882844

[6] Tubo NJ, Pagán AJ, Taylor JJ, Nelson RW, Linehan JL, et al (2013) Single naive CD4+ T cells from a diverse repertoire produce different effector cell types during infection. Cell 153: 785–796. 23663778 10.1016/j.cell.2013.04.007 PMC3766899

[7] Plumlee C, Sheridan B, Cicek B, Lefrançois L (2013) Environmental cues dictate the fate of individual CD8+ T cells responding to infection. Immunity 39: 347–356. 23932571 10.1016/j.immuni.2013.07.014 PMC3817618

[8] Chang JT, Palanivel VR, Kinjyo I, Schambach F, Intlekofer AM, et al (2007) Asymmetric T lymphocyte division in the initiation of adaptive immune responses. Science 315: 1687–1691. 17332376 10.1126/science.1139393

[9] Oliaro J, Van Ham V, Sacirbegovic F, Pasam A, Bomzon Ze, et al (2010) Asymmetric cell division of T cells upon antigen presentation uses multiple conserved mechanisms. The Journal of Immunology 185: 367–375. 20530266 10.4049/jimmunol.0903627 PMC3739982

[10] Chang JT, Ciocca ML, Kinjyo I, Palanivel VR, McClurkin CE, et al (2011) Asymmetric proteasome segregation as a mechanism for unequal partitioning of the transcription factor T-bet during T lymphocyte division. Immunity 34: 492–504. 21497118 10.1016/j.immuni.2011.03.017 PMC3088519

[11] Miller MJ, Safrina O, Parker I, Cahalan MD (2004) Imaging the single cell dynamics of CD4+ T cell activation by dendritic cells in lymph nodes. The Journal of experimental medicine 200: 847–856. 15466619 10.1084/jem.20041236 PMC2213293

[12] Miller MJ, Wei SH, Parker I, Cahalan MD (2002) Two-photon imaging of lymphocyte motility and antigen response in intact lymph node. Science 296: 1869–1873. 12016203 10.1126/science.1070051

[13] Mempel TR, Henrickson SE, von Andrian UH (2004) T-cell priming by dendritic cells in lymph nodes occurs in three distinct phases. Nature 427: 154–159. 14712275 10.1038/nature02238

[14] Scholer A, Hugues S, Boissonnas A, Fetler L, Amigorena S (2008) Intercellular Adhesion Molecule-1-Dependent Stable Interactions between T Cells and Dendritic Cells Determine CD8+ T Cell Memory. Immunity 28: 258–270. 18275834 10.1016/j.immuni.2007.12.016

[15] Dustin ML, Bromley SK, Kan ZY, Peterson DA, Unanue ER (1997) Antigen receptor engagement delivers a stop signal to migrating T lymphocytes. Proceedings of the National Academy of Sciences of the United States of America 94: 3909–3913. 9108078 10.1073/pnas.94.8.3909 PMC20541

[16] Monks C, Freiberg B, Kupfer H, Sciaky N, Kupfer A (1998) Three-dimensional segregation of supramolecular activation clusters in T cells. Nature 395: 82. 9738502 10.1038/25764

[17] Grakoui A, Bromley SK, Sumen C, Davis MM, Shaw AS, et al (1999) The immunological synapse: A molecular machine controlling T cell activation. Science 285: 221–227. 10398592 10.1126/science.285.5425.221

[18] Huppa JB, Davis MM (2003) T-cell-antigen recognition and the immunological synapse. Nature Reviews Immunology 3: 973–983. 10.1038/nri1245 14647479

[19] Chang JT (2012) Polarity and lymphocyte fate determination. Current Opinion in Cell Biology 24: 526–533. 22658837 10.1016/j.ceb.2012.05.002 PMC3425726

[20] Irvine DJ, Doh J (2007) Synthetic surfaces as artificial antigen presenting cells in the study of T cell receptor triggering and immunological synapse formation. Seminars in Immunology 19: 245–254. 17398113 10.1016/j.smim.2007.02.011

[21] Jung HR, Choi JC, Cho W, Doh J (2012) Microfabricated platforms to modulate and monitor T cell synapse assembly. Wiley Interdisciplinary Reviews: Nanomedicine and Nanobiotechnology. 10.1002/wnan.1182 22927231

[22] Doh J, Irvine DJ (2006) Immunological synapse arrays: Patterned protein surfaces that modulate immunological synapse structure formation in T cells. Proceedings of the National Academy of Sciences of the United States of America 103: 5700–5705. 16585528 10.1073/pnas.0509404103 PMC1458636

[23] Dustin ML (2004) Stop and go traffic to tune T cell responses. Immunity 21: 305–314. 15357942 10.1016/j.immuni.2004.08.016

[24] Shen K, Thomas VK, Dustin ML, Kam LC (2008) Micropatterning of costimulatory ligands enhances CD4+ T cell function. Proceedings of the National Academy of Sciences of the United States of America 105: 7791–7796. 18505845 10.1073/pnas.0710295105 PMC2409411

[25] Irvine DJ, Doh J, Huang B (2007) Patterned surfaces as tools to study ligand recognition and synapse formation by T cells. Current Opinion in Immunology 19: 463–469. 17616382 10.1016/j.coi.2007.05.003

[26] Doh J, Kim M, Krummel MF (2010) Cell-laden microwells for the study of multicellularity in lymphocyte fate decisions. Biomaterials 31: 3422–3428. 20117834 10.1016/j.biomaterials.2010.01.018

[27] Sowinski S, Jolly C, Berninghausen O, Purbhoo MA, Chauveau A, et al (2008) Membrane nanotubes physically connect T cells over long distances presenting a novel route for HIV-1 transmission. Nature Cell Biology 10: 211–219. 18193035 10.1038/ncb1682

[28] Mukai A, Mizuno E, Kobayashi K, Matsumoto M, Nakayama KI, et al (2008) Dynamic regulation of ubiquitylation and deubiquitylation at the central spindle during cytokinesis. Journal of Cell Science 121: 1325–1333. 18388320 10.1242/jcs.027417

[29] Bisikirska B, Colgan J, Luban J, Bluestone JA, Herold KC (2005) TCR stimulation with modified anti-CD3 mAb expands CD8+ T cell population and induces CD8+CD25+ Tregs. Journal of Clinical Investigation 115: 2904–2913. 16167085 10.1172/JCI23961 PMC1201661

[30] Lai YP, Lin CC, Liao WJ, Tang CY, Chen SC (2009) CD4+ T cell-derived IL-2 signals during early priming advances primary CD8+ T cell responses. PLoS ONE 4: e7766. 19901991 10.1371/journal.pone.0007766 PMC2770320

[31] King C, Koehli S, Hausmann B, Schmaler M, Zehn D, et al (2012) T Cell Affinity Regulates Asymmetric Division, Effector Cell Differentiation, and Tissue Pathology. Immunity 37: 709–720. 23084359 10.1016/j.immuni.2012.06.021 PMC3622938

